# Investigating the Interplay of Toxic Metals and Essential Elements in Cardiovascular Disease

**DOI:** 10.3390/jox15030068

**Published:** 2025-05-09

**Authors:** Aderonke Gbemi Adetunji, Emmanuel Obeng-Gyasi

**Affiliations:** 1 Department of Built Environment, North Carolina A&T State University, Greensboro, NC 27411, USA; 2 Environmental Health and Disease Laboratory, North Carolina A&T State University, Greensboro, NC 27411, USA

**Keywords:** mixtures, trace elements, toxic metals, cardiovascular, cholesterol, blood pressure

## Abstract

Cardiovascular diseases (CVDs) are the leading cause of mortality globally, accounting for approximately one-third of all deaths. Exposure to toxic metals poses significant risks to cardiovascular health, contributing to the development of CVDs. Essential elements are crucial for maintaining cardiovascular function; however, imbalances or deficiencies in these elements can exacerbate the risk and progression of CVDs. Understanding the interactions between toxic metals and essential elements is crucial for elucidating their impact on cardiovascular health. This study aims to examine the individual and combined effects of toxic metals—lead (Pb), cadmium (Cd), and mercury (Hg)—along with essential elements—manganese (Mn), iron (Fe), and selenium (Se)—on CVDs. We explored the effects of toxic metals and essential elements using data from the National Health and Nutrition Examination Survey (NHANES, 2017–2018). We conducted descriptive analyses and applied advanced statistical methods, including Bayesian kernel machine regression (BKMR), weighted quantile sum regression (WQSR), and quantile g-computation, to assess the associations between these toxic metals and essential elements on key cardiovascular-related biomarkers. The results revealed distinct patterns of influence across the toxic metals and essential elements. Spearman correlation showed a stronger association among toxic metals than essential elements. Bayesian kernel machine regression (BKMR) and posterior inclusion probability (PIP) analysis identified lead, mercury, iron, and selenium as key contributors to CVD risk, with lead strongly linked to high-density lipoprotein (HDL), diastolic blood pressure (DBP), and systolic blood pressure (SBP). Selenium was linked to low-density lipoprotein (LDL) cholesterol and non-high-density lipoprotein (non-HDL) cholesterol. Univariate and bivariate analyses confirmed lead and mercury’s strong associations with triglycerides and blood pressure, while lead, selenium, and iron were linked to different cholesterol outcomes. Single-variable analysis revealed an interaction between individual exposures and combined exposures. The overall exposure effect assessing the impact of all exposures combined on CVD markers revealed a steady positive association with triglycerides, total cholesterol, LDL, non-HDL cholesterol, and DBP, with HDL and SBP increasing from the 65th percentile. Quantile g-computation and WQSR confirmed lead’s consistent positive association across all outcomes, with variations among other toxic metals and essential elements. In conclusion, our study suggests that toxic metals and essential elements are important factors in CVD outcomes, with different metals and elements associated with variations in specific biomarkers.

## 1. Introduction

### 1.1. Overview of Cardiovascular Disease

Cardiovascular diseases (CVDs) encompass a range of disorders affecting the heart and blood vessels, including coronary heart disease, cerebrovascular disease, rheumatic heart disease, and various other related conditions [1,2]. CVDs are the primary cause of mortality around the world, resulting in around 17.9 million deaths, which accounts for almost 31% of all deaths worldwide [1]. Over 80% of CVD deaths result from heart attacks and strokes, with a third of these deaths happening prematurely in individuals below the age of 70 [3]. The occurrence of CVDs differs across regions, with more than three-quarters of CVD-related deaths happening in low- and middle-income nations [4,5].

In the United States, CVD presents a substantial public health challenge. Approximately 697,000 individuals in the United States died of heart disease in 2020. The deaths from heart disease represent one-fifth of all deaths in the United States [6]. Coronary heart disease is prevalent as the primary form of CVD dysfunction, impacting approximately 20.1 million adults aged 20 and older in the United States [6]. More than 795,000 individuals in the United States experience either a new or recurrent stroke each year. Between 2018 and 2019, the expenses associated with stroke in the United States amounted to almost USD 56.5 billion [7]. This sum encompasses healthcare service costs, medications utilized for stroke treatment, and lost productivity due to missed workdays [7]. Racial and ethnic minority groups experience a disproportionate burden of cardiovascular diseases, with Black, Hispanic, and Native American populations exhibiting higher prevalence rates compared to white individuals [7]. Despite reductions in overall CVD mortality rates in recent years, it has continued to be the leading cause of death in the United States with its significant impact on healthcare expenses and productivity. It is crucial for policymakers, healthcare professionals, and communities to tackle the significant burden of CVDs and enhance the health and welfare of all Americans.

### 1.2. Common Causes and Risk Factors for Cardiovascular Disease

A variety of factors contribute to the development of CVDs; some of these factors include an unhealthy diet, physical inactivity, family history, and environmental factors, with environmental and dietary influences playing significant roles among them. Unhealthy dietary habits, characterized by excessive consumption of saturated fats, trans fats, sodium, processed foods, and refined carbohydrates, along with a deficiency of nutrient-rich foods such as fruits and vegetables, markedly elevate the likelihood of developing CVDs. Conversely, diets abundant in fruits, vegetables, whole grains, and lean sources of fats and proteins are linked to a decreased risk of CVDs [8], suggesting the importance of adhering to dietary guidelines [9]. Therefore, diet is an important factor that determines the cardiovascular health of an individual.

Physical inactivity and sedentary behaviors, such as prolonged sitting, minimal physical activity, and limited sleep, among others, are significant risk factors for CVDs. However, engaging in regular exercise contributes to weight management, lowers blood pressure, and improves cholesterol levels, ultimately fostering overall health [10]. On the other hand, obesity, which is characterized by excessive accumulation of fat in the body, especially in the abdominal region, is a well-established risk factor for CVDs. Obesity can promote various metabolic abnormalities that contribute to the development of CVDs including hypertension, dyslipidemia (abnormal lipid levels), and insulin resistance, which can lead to type 2 diabetes [11]. Interventions targeting the reduction in high blood pressure, cholesterol, and glucose levels could potentially address roughly half of the excessive risk of coronary heart disease associated with an elevated BMI and approximately three-quarters of the excess risk of stroke. Maintaining an optimal body weight is crucial to fully realize these benefits [12].

Environmental factors including exposure to toxic metals and secondhand smoke, occupational hazards, air pollution, noise pollution, contaminated drinking water, and psychosocial stressors have been shown to be associated with the development of CVDs through various mechanisms, including oxidative stress, inflammation, and endothelial dysfunction [13]. Exposure to toxic metals like lead and cadmium and exposure to secondhand smoke poses risks to cardiovascular health. These metals, harmful chemicals, and particulate matter from secondhand smoke have the potential to build up in the body, contributing to the onset of hypertension, atherosclerosis, and other cardiovascular diseases [13].

Ethnic variations in the prevalence of CVDs have been observed; according to the Heart Disease and Stroke Statistics 2018 update, the prevalence of heart disease varies across different ethnic groups, with 11.1% among non-Hispanic whites, 10.3% among Black or African Americans, 7.8% among Hispanic or Latinos, 6.0% among Asians, 13.7% among American Indians or Alaska Natives, and 19.1% among Native Hawaiians or other Pacific Islanders [2]. Ethnic minority populations encounter heightened cardiovascular disease risks stemming from socioeconomic challenges, including lower income and limited access to quality healthcare. These factors can impede the prompt identification and treatment of risk factors [14].

The interplay of diets, physical activity, environmental factors and ethnicity and other risk factors, alongside other variables, contribute to an individual’s susceptibility to CVDs. Hence, it is imperative to target these modifiable risk factors through public health initiatives and personal behavior modifications to prevent and effectively manage cardiovascular conditions.

### 1.3. Importance of Metals and Essential Metals in Cardiovascular Diseases

Toxic metals, often referred to as heavy metals, encompass a group of naturally occurring metallic elements such as lead, cadmium, and mercury, characterized by their high atomic weight and densities. They are known for their detrimental impacts on both human health and the surrounding environment [15]. Toxic metals have various pathways to enter the body, including ingestion via contaminated food or water, inhalation as particles in air pollution or in certain occupational environments like mining, and absorption through the skin when it comes into contact with contaminated water, soil, or other substances. After entering the body, these harmful metals have the potential to accumulate in different organs and tissues, resulting in a variety of negative health outcomes, including CVDs [16].

Environmental metals such as lead (Pb), cadmium (Cd), and mercury (Hg) rank among the top ten most toxic substances posing significant risks to public health due to their high degree of toxicity and widespread presence [15]. Their extensive use across industrial, domestic, agricultural, medical, and technological sectors has contributed to their broad environmental distribution, heightening concerns about their potential impacts on both human health and the ecosystem [15]. Studies have explored the effect of these toxic metals on cardiovascular health. In a cross-sectional analysis of data from the Malmö Diet and Cancer Study cardiovascular cohort (MDCS-CC), recruited between 1991 and 1994, findings indicated an association between blood lead (Pb) levels and the presence of atherosclerotic plaque in the carotid artery [17]. In another study using NHANES data (1999–2016), elevated serum cadmium levels were linked to increased lipid and inflammatory markers, including triglycerides, total cholesterol, white blood cells (WBCs), and C-reactive protein (CRP), suggesting that cadmium may raise cardiovascular disease risk by promoting lipid elevation and inflammation [18]. Given these findings, reducing exposure to toxic metals and addressing their cardiovascular impacts are critical steps for improving public health and preventing cardiovascular disease.

Essential elements, often referred to as mineral nutrients, are inorganic compounds required for numerous physiological functions within the human body. These essential elements cannot be internally synthesized and must be obtained through dietary intake or supplementation. These essential elements perform crucial functions in a multitude of biological processes, encompassing enzyme activation, the formation of tissue and bone structures, maintenance of fluid balance, nerve signaling, muscle function, immune response, and protection against oxidative stress [19]. Some of these essential elements include iron (Fe), manganese (Mn), and selenium (Se). These elements also serve as critical cofactors for many enzymes involved in maintaining cardiovascular health. However, exposure to toxic metals can interfere with the absorption, distribution, and utilization of essential elements, disrupting this delicate balance. Such interference can promote oxidative stress and inflammation, both of which are key contributors to the onset and progression of cardiovascular diseases [20]. Imbalance or deficiencies in essential elements can result in diverse health issues, including CVDs, underscoring the importance of maintaining adequate intake through a balanced diet or supplementation to maintain overall health and avoid disorders related to minerals [21,22].

Studies have investigated the relationship between Mn, Fe, and Se and cardiovascular health. Using NHANES data from 2017 to 2023, elevated blood levels of Mn and Se were associated with a higher prevalence of hepatic steatosis in adolescents, suggesting a potential role of these trace elements in its development [23]. Additionally, another study reported a correlation between blood iron levels and the presence of coronary atherosclerosis [24].

Existing research has explored the association between exposure to toxic metals and CVD risk, as well as essential elements and CVDs, yet the potential synergistic effects of toxic metals and essential elements on CVD risk remains underexplored. The complex relationship between toxic metals and essential elements within the cardiovascular system significantly influences the onset and progression of CVDs. Gaining insight into these intricate interactions is paramount for formulating efficient approaches to prevent and address CVDs associated with imbalances in metal levels. This understanding is pivotal for devising strategic interventions to safeguard cardiovascular health in the face of metal-related challenges. Hence, the aim of this research is to evaluate the independent and collective impacts of toxic metals ((Pb), (Cd), (Hg)) and essential elements ((Mn), (Fe), (Se)) on the risk of CVDs by analyzing data derived from the National Health and Nutrition Examination Survey (NHANES).

## 2. Materials and Methods

### 2.1. Participants Sampling and Description

This study utilized data from the National Health and Nutrition Examination Survey (NHANES) for the years 2017–2018. NHANES is a cross-sectional, multiphase survey designed to assess the nutritional status and overall health of a nationally representative sample of non-institutionalized individuals in the United States. This dataset represents individuals residing in all 50 U.S. states and the District of Columbia. Conducted by the U.S. Centers for Disease Control and Prevention (CDC), the data are collected in two-year cycles and consist of multi-year, stratified, multi-stage, and clustered samples. Informed consent was obtained from all participants who underwent physical examinations and interviews. Blood samples were collected from the participants and sent to a laboratory for analysis. The survey protocols were approved by the Institutional Review Board at the National Center for Health Statistics (NCHS), part of the CDC.

Additionally, demographic factors such as age, sex, and ethnicity were collected through household interviews conducted using a computer-assisted personal interview (CAPI) system, ensuring accurate and reliable data collection. These interviews were administered in various languages to accommodate the diverse backgrounds of the participants.

### 2.2. Essential Element and Metal Quantification

Essential elements including Fe, Se, and Mn were measured using the Roche Cobas 6000 (c501 module) method. These assays were conducted at the University of Minnesota Advanced Research and Diagnostic Laboratory (ARDL) under the National Center for Environmental Health (NCEH) of the CDC’s Division of Laboratory Sciences. Detailed protocols for specimen collection, storage, and analysis are provided in the NHANES Laboratory Procedures Manual [25]. The toxic metal (Pb, Cd, and Hg) content in whole blood specimens was directly measured using mass spectrometry following a straightforward dilution sample preparation step (ICP-MS; CDC method no. ITB0001A). Detailed laboratory procedures can be accessed through the CDC [26].

### 2.3. Measuring Cardiovascular Variables

Blood pressure measurements were taken while the participants were seated comfortably after a five-minute rest period. The assessments were conducted using a mercury sphygmomanometer, strictly adhering to the standardized blood pressure measurement guidelines established by the American Heart Association [26].

An analysis of lipid biomarkers was carried out using the Beckman Synchron LX20 and Beckman UniCel^®^ DxC800 Synchron instruments at Collaborative Laboratory Services in Brea, CA, USA. Additionally, the Roche Modular P chemistry analyzer was used at the University of Minnesota in Minneapolis, MN, USA. LDL cholesterol levels were calculated with the use of the Friedewald equation. The determination of plasma levels for fasting and 2 h glucose was accomplished through a hexokinase assay, which was conducted on a Roche/Hitachi 911 Analyzer and a Roche Modular P Chemistry Analyzer(Roche Diagnostics GmbH, Mannheim, Germany).

### 2.4. Statistical Analysis

#### 2.4.1. Descriptive Statistics

In this study, we used descriptive statistics to summarize key variables in the dataset, such as sex, age, and ethnicity.

#### 2.4.2. Bayesian Kernel Machine Regression (BKMR)

The study utilized the BKMR modeling approach to evaluate the collective impact of essential elements and toxic metals on variables associated with cardiovascular health. BKMR is a widely adopted method by numerous researchers to assess the combined effect of multiple pollutants [27,28,29] on various health outcomes due to its ability to effectively model non-linear and non-additive relationships. This technique is adept at capturing complex interactions and dependencies within the data [30,31] and provides a more precise evaluation of the combined impact of contaminants on a specific outcome of interest.

The model employed in our study is as follows:

g(*µ_i_*) = *h*(*z_i_*_1_, . . . . . . ., *z_iM_*) + *β*X*_i_*; *i* = 1, . . . ., n (1)

where g is the monotonic link function, *µ_i_* = *E(Yi)*; *h* is the flexible kernel function of exposures *z_i_*_1_, . . . . . . ., *z_iM_* with x being the vector of covariates with a linear association with CVD; and *β* represents a vector of associated coefficients [31].

The predictors (Z) are the essential elements and toxic metal variables, and h(.) represents the exposure–response function. The BKMR model uses multiple representations to explore the patterns of association and interaction between essential elements, toxic metals, and CVD risk. This approach provides insights into both univariate and bivariate relationships, interactions, overall effects, and single-variable effects and interactions [31,32]. It helps address the complex effects of exposure to environmental mixtures by considering various scenarios of exposure–response functions.

#### 2.4.3. Weighted Quantile Sum Regression (WQSR)

Weighted quantile sum (WQS) regression is a statistical methodology developed for multivariate regression in high-dimensional datasets. The approach comprises two primary phases, namely a training phase, during which the weights are estimated, and a validation phase, where the regression model is implemented using the previously computed weights. The mathematical formulation of the WQS model is expressed as follows:

(2) $$g\left(\mu \right)=\beta_{0}+\beta 1\left(\sum_{i=1}^{c}w_{i}q_{i}\right)+z’\varphi$$

where g represents the link function, consistent with generalized linear models, and μ denotes the mean of the outcome variable. The term qi corresponds to the quantile of the ith component and wi represents the associated weight, which is estimated during model fitting. Additionally, z′ is the vector of covariates, and φ is the parameter vector corresponding to these covariates. The term $\sum_{i=1}^{c}w_{i}q_{i}^{q}$ denotes the index that assigns weights to the components within the mixture and aggregates their contributions. To construct the model, the dataset was partitioned into two subsets, comprising a training set for estimating weights and a validation set for evaluating the statistical significance of the resulting WQS index. The weights were estimated using a bootstrap procedure and constrained to sum to one while remaining within the bounds of zero and one: $\sum_{i=1}^{c}w_{i}=1$ and 0 ≤ wi ≤ 1. For each bootstrap sample, a dataset was generated by sampling with a replacement from the training dataset. The model parameters, defined as θ = (β_0_,β_1_,w_1_,…,w_c_,φ), were estimated using an optimization algorithm, with the log-likelihood function serving as the objective criterion.

(3) $$\hat{\theta }WQS=argmax\theta \left[l\left(\theta ;y\right)+\lambda \;\left(\sum_{i=1}^{c}w_{i}-1\right)\;\right]$$

where l(θ;y) represents the log-likelihood function and λ denotes the Lagrangian coefficient corresponding to the equality constraint, ensuring that the weights sum to one. Additionally, an inequality constraint is imposed to maintain the bounds 0 ≤ wi ≤1. After estimating the weights, the model is applied to determine the regression coefficients at each stage of the ensemble process. Upon completion of the bootstrap ensemble, the estimated weights are aggregated by computing their average across all bootstrap samples, resulting in the final WQS index:

(4) $$WQS=\sum_{i=1}^{c}{\bar{w}}_{i}qᵢ$$

where

(5) $${\bar{w}}_{i}=\frac{1}{\sum_{b=1}^{B}f\left(\beta_{i}\left(b\right)\right)}\sum_{b=1}^{B}w_{i}\left(b\right)f\left(\beta_{i}\left(b\right)\right)\;and\;f\left(\beta_{i}\left(b\right)\right)\;$$

is a signal function. The weights were first estimated using a training set and subsequently applied to construct a WQS index within a validation set. This index is then utilized to assess the association between the mixture and the health outcome within a standard generalized linear model as follows:

(6) $$g\left(\mu \right)=\beta_{0}+\beta 1\;WQS+z’\varphi$$

Given the model’s structure, the association between the dependent variable and the WQS index was constrained to either a positive or negative direction. This design ensures that the model remains inherently one-directional, assessing mixture effects that are either positively or negatively linked to the outcome. To account for associations in both directions, the analysis was conducted twice, one for each direction.

The one-directional nature of the index mitigated the risk of a reversal paradox, which could arise in the presence of highly correlated variables. Additionally, the bootstrap procedure enhanced the identification of key contributors.

Following the fitting of the final model, the significance of β1 was evaluated to assess the presence of an association between the WQS index and the outcome. If the coefficient significantly deviated from zero, the weights were analyzed, with the highest values indicating the key components contributing to the observed association.

In this study, the WQS index for heavy metal and essential element exposure was developed using quartiles of blood metal and essential element concentrations. The dataset was partitioned, with 40% allocated for testing and the remaining 60% designated for validation. The WQS model was further adjusted for potential confounders, including age, sex, race/ethnicity, BMI, income, smoking status, and alcohol consumption.

#### 2.4.4. Quantile g-Computation

Quantile g-computation enhances the estimation of exposure effects by accounting for non-linear and non-additive relationships. The initial step involves transforming the exposures (Xj) into their quantized counterparts (Xqj). Subsequently, a linear model is fitted to assess the association:

(7) $$Y_{i}=\beta_{0}+\sum_{j=1}^{d}\beta_{j}X_{\;ji}^{\;q}+\beta_{j}Z+\varepsilon_{i}$$

In this model, $Y_{i}$ is the outcome variable for individual i, such as a health measure or biomarker level. The term $\beta_{0}$ is the intercept, representing the expected outcome when all predictors are at their reference levels. The summation term, $\sum_{j=1}^{d}\beta_{j}X_{\;ji}^{\;q}$), captures the cumulative effect of d envronmental exposures, where each exposure $X_{\;ji}^{\;q}$ has been transformed into quantiles (denoted by the superscript q). This quantile transformation allows the model to better capture non-linear exposure–outcome relationships. The coefficient $\beta_{j}$ represents the effect size associated with each exposure. Additionally, $\beta_{j}Z$ accounts for the influence of covariates Z, which may include demographic factors, socioeconomic status, or other confounders that could affect the outcome. Lastly, $\varepsilon_{i}$ represents the error term, capturing unexplained variability in the outcome.

In quantile g-computation, if directional homogeneity is present, ψ is defined as $\sum_{j=1}^{d}\beta_{j}$, where $\beta_{j}$ represents the effect size for exposure *j*. The weights for each exposure, indexed by *k*, are calculated as $wk=\beta_{k}/\sum_{j}^{d}\beta_{j}$, ensuring that the weights collectively sum to 1.0.

When directional homogeneity is not met, quantile g-computation adjusts the weight definitions to distinguish between positive and negative weights. These weights represent the proportion of the total positive or negative partial effect attributable to a specific exposure. Additionally, the positive and negative weights are constrained to sum to 1.0.

This method provides a versatile approach suitable for different data types and outcomes. We implemented quantile g-computation to explore the associations between essential elements and toxic metals on CVD risk and corresponding weights for each heavy metal and essential element. Quantile g-computation was used to estimate the change in CVD risk for a simultaneous one-quantile increase in essential elements and toxic metals [33].

All analyses in this study were conducted using R (version 4.2.1; R Foundation for Statistical Computing, Vienna, Austria), with a significance level set to 0.05 for non-Bayesian analysis.

## 3. Results

### 3.1. Descriptive Analysis of Sex, Ethnicity, and Age

Table 1a,b summarizes the descriptive statistics for our study; the dataset demographics revealed distinct age distributions across sex and racial/ethnic groups. Males and females had average ages of 37 and 39 years, respectively, while different ethnic subgroups showed varying average ages, with non-Hispanic whites representing an older demographic and Mexican Americans representing a younger demographic. These findings highlight variability across groups and provide critical context for interpreting subsequent analyses [34].

### 3.2. Correlation Between Variables of Interest

**Figure 1:**
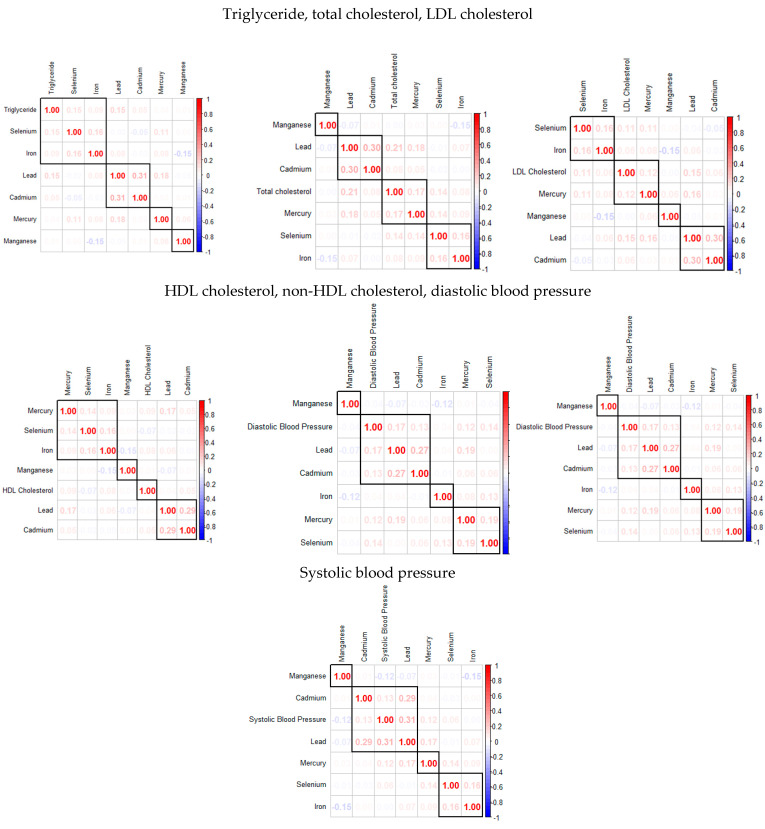
The Spearman correlation analysis among the study’s exposure variables.

Figure 1 examines the correlations among the key study variables, including metals, essential elements, and individual cardiovascular disease biomarkers. The analysis reveals that these groups exhibit varying degrees of correlation within themselves. Specifically, toxic metals tend to have stronger inter-correlations, and there is an inverse relationship observed between certain toxic metals and essential elements.

### 3.3. BKMR Analysis

#### 3.3.1. Posterior Inclusion Probabilities (PIPs)

Table 2 displays the PIP for each toxic metal and essential element in relation to CVD health biomarkers. The PIP represents the probability that a particular contaminant significantly contributes to the variation observed in a specific health outcome. For instance, a PIP of 0.9838 for selenium with respect to triglyceride levels indicates a very high probability that selenium plays an influential role in affecting triglyceride levels. Conversely, a PIP of 0.3022 for lead suggests a comparatively lesser influence on triglyceride levels. Furthermore, a PIP of 1.000 for lead, mercury, selenium, and iron in relation to total cholesterol indicates a strong likelihood that these elements are key contributors to the variability in total cholesterol. At the same time, cadmium, with a PIP of 0.0000, likely has no impact.

#### 3.3.2. BKMR Models Showing PIP for Exposure to Metals and Essential Elements and by Health Outcome

Table 3, Table 4, Table 5, Table 6, Table 7, Table 8 and Table 9 present the PIPs derived from the hierarchical BKMR analysis for triglycerides, total cholesterol, LDL cholesterol, HDL cholesterol, non-HDL cholesterol, diastolic blood pressure, and systolic blood pressure. This analysis employs a hierarchical structure to categorize exposure variables into distinct groups, providing both group PIP and conditional PIP (cond PIP) values. The group PIP quantifies the probability that an exposure group is associated with the outcome, reflecting its overall relevance within the model. The conditional PIP, in contrast, represents the probability that a specific exposure within a selected group is included in the model, given that the group itself is relevant. This metric facilitates the identification of key exposures within a mixture by assessing their individual contributions conditionally, based on the selection of their respective group. By incorporating both group PIPs and conditional PIPs, the hierarchical BKMR framework enables a more nuanced evaluation of exposure mixtures, distinguishing broad group-level associations from specific influential exposures within each category.

In the hierarchical BKMR results, the group column categorizes variables into two groups: Group 1 includes toxic metals such as Pb, Cd, and Mg, while Group 2 includes essential elements like Se, Mn, and Fe.

For triglyceride levels, Group 1 (toxic metals) shows high group PIP values (0.8900), with mercury standing out with the highest conditional PIP of 1.0000, suggesting it has a dominant influence on triglyceride levels within this group. In Group 2 (essential elements), all variables also have high group PIP values (0.9852). Among these, selenium has the highest conditional PIP value (0.7353), indicating a significant impact on triglyceride levels.

For total cholesterol, the toxic metals in Group 1 and essential elements in Group 2 have the same Group PIP value of one. However, lead and selenium have conditional PIP values of one within their group, with other exposures in the group having conditional PIP values of 0. This suggests that lead and selenium have the strongest impact on total cholesterol compared to the other exposures in their respective group.

For LDL cholesterol, the toxic metals in Group 1 and essential elements in Group 2 have the same group PIP value of one; however, lead and selenium have conditional PIP values of 0.9556 and 0.8156, respectively, suggesting these exposures have the most impact in their respective group.

For HDL cholesterol, these results suggest that while both toxic metals and essential elements are relevant with group PIPs of one each, lead and iron stand out as particularly important exposures in explaining variations in HDL cholesterol.

For non-HDL cholesterol and diastolic blood pressure, these results suggest that while toxic metals and essential elements are relevant with group PIPs of one each, lead and selenium are important in explaining variations in non-HDL cholesterol and total cholesterol.

For systolic blood pressure, these results suggest that while toxic metals and essential elements are relevant, lead and manganese stand out as particularly important exposures in explaining variations in systolic blood pressure.

#### 3.3.3. Univariate Association of Toxic Metals and Essential Elements with CVD

**Figure 2:**
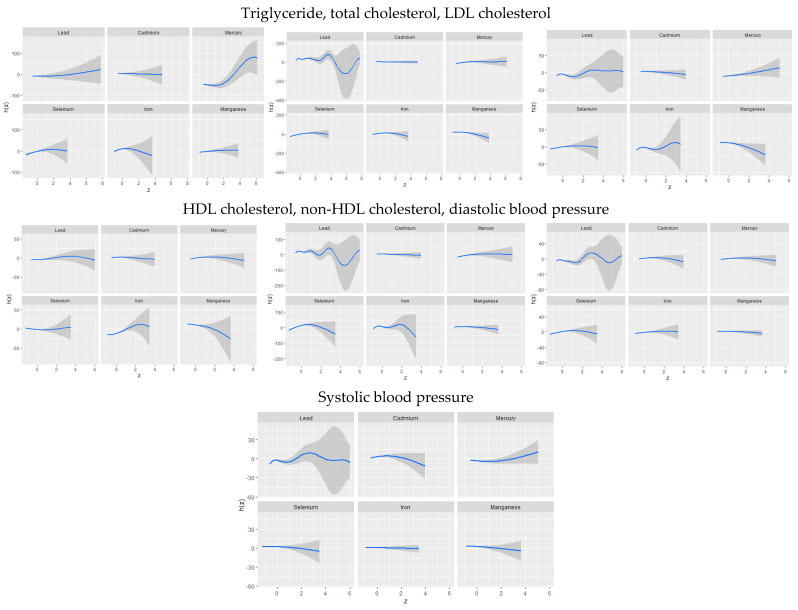
Univariate exposure–response function and 95% credible interval for the association between single metal/essential element exposure with CVD risk when other exposures are fixed at the median. Adjusted for age, sex, race/ethnicity, BMI, income, smoking status, and alcohol use.

The univariate approach visually examines the individual effect of lead, cadmium, mercury, iron, manganese, and selenium on CVD risk outcomes when the other exposures are fixed at the median and the covariates are adjusted for (Figure 2). The gray bands represent 95% credible intervals.

#### 3.3.4. Bivariate Exposure–Response Function of Toxic Metals and Essential Elements with CVD

**Figure 3:**
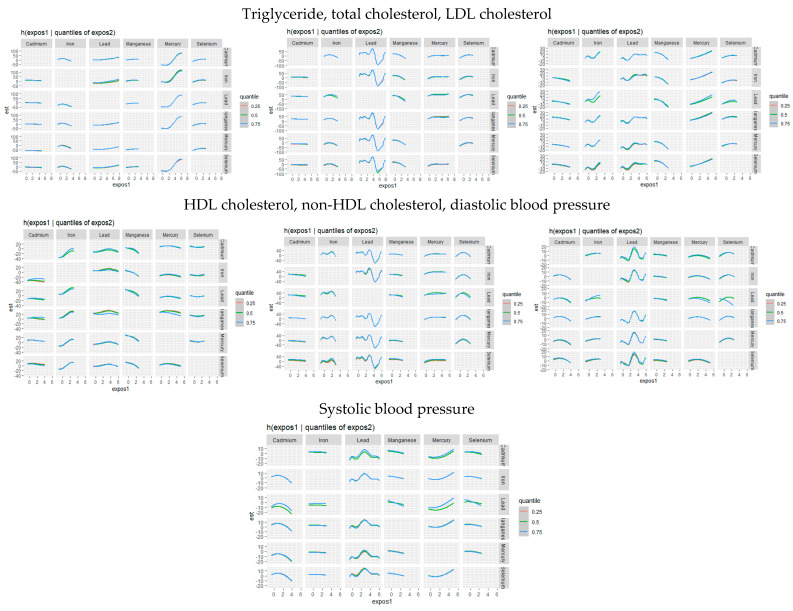
Bivariate exposure–response function of toxic metal and/or essential element with CVD risk when other exposures are fixed at the median. Adjusted for age, sex, race/ethnicity, BMI, income, smoking status, and alcohol use.

Figure 3 presents an analysis of the association between individual metals and essential elements on cardiovascular disease CVD by fixing a second metal or essential element at different quantiles (25th in red, 50th in green, and 75th in blue) while holding other metals and essential elements at their median values. These models were adjusted for relevant covariates. The *x*-axis, labeled “expos1”, shows the levels of one exposure, while the *y*-axis, labeled “est”, indicates the estimated effect on CVD levels. Each column and row on the plots represents a different exposure.

#### 3.3.5. Single-Variable Effects of Toxic Metals and Essential Elements on Cardiovascular-Related Markers

**Figure 4:**
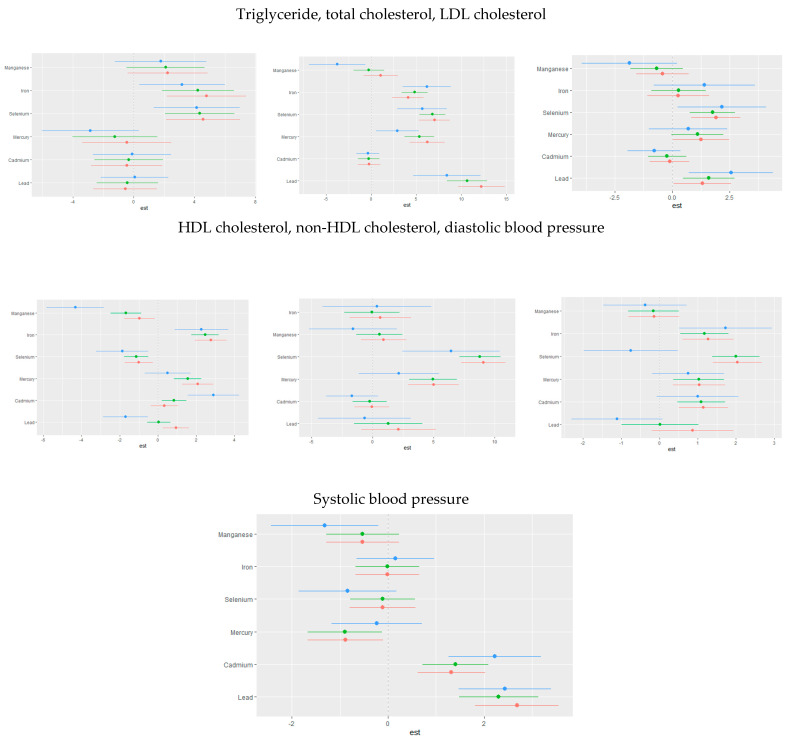
Single-exposure attributable effects (95% credible intervals) representing the change in CVD risk associated with a change in a specific exposure (toxic metal/essential element) from its 25th to its 75th percentile, with all other exposures fixed at quantiles of 0.25 (red), 0.50 (green), or 0.75 (blue) quantile. Adjusted for age, sex, race/ethnicity, BMI, income, smoking status, and alcohol use.

The univariate effect emphasizes the impact of a single predictor across various quantiles. Figure 4 shows the single variable impacts of toxic metals and critical elements on certain CVD-related biomarkers at the 25th (red), 50th (green), and 75th (blue) quantiles. Analyzing these single-variable impacts allows for a clearer understanding of the individual influence of each exposure factor on CVD outcome.

#### 3.3.6. Single-Variable Interaction Terms of Toxic Metals and Essential Elements on Individual CVD-Related Markers

**Figure 5:**
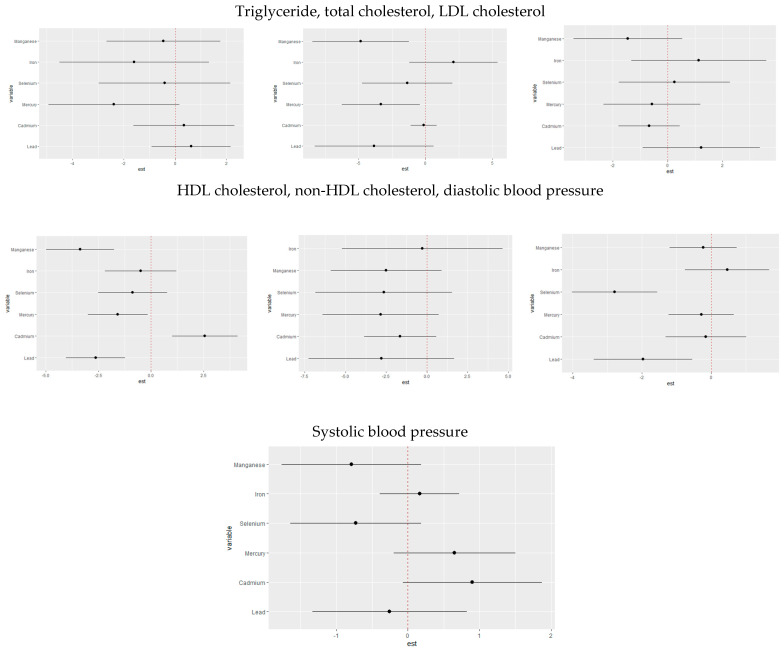
Single-variable interaction terms for toxic metals/essential elements, comparing the effect of each toxic metal/essential element exposure when all other toxic metals/essential elements are fixed at the 75th percentile compared to the 25th percentile. Adjusted for age, sex, race/ethnicity, BMI, income, smoking status, and alcohol use.

We investigated the interaction effects between exposures to toxic metals and essential elements. Our analysis estimates the probability of interaction inclusion for each variable pair, identifying cases where their combined effect adds significant explanatory power to the CVD outcome variable beyond their individual effects. Figure 5 illustrates the single-variable effect of an individual toxic metal or essential element when all other metals and essential elements are held at either the 25th or 75th quantile.

#### 3.3.7. Overall Risk Summary of CVD Levels in Relation to Exposure Percentiles

**Figure 6:**
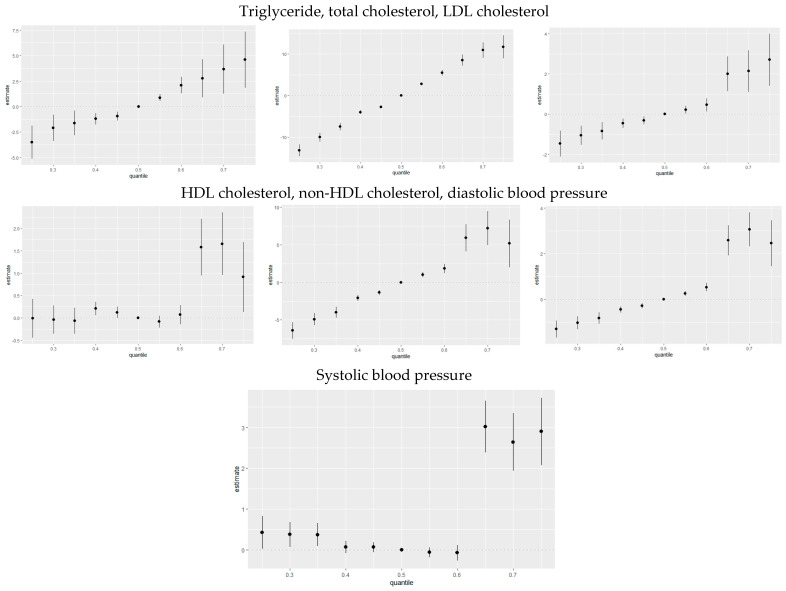
The overall health effects of the exposures calculated by comparing the value of h when all of the predictors are at quantiles between 0.025 to 0.75 as compared to when all of them are at their 0.50 quantile percentile.

Figure 6 quantifies the cumulative effect of all exposures or mixtures. Exposures are held constant at various percentiles ranging from the 25th to the 75th percentile, increasing by increments of five, with the 50th percentile (median) serving as a reference for comparison. As shown in the plots, the estimation risk of CVD for all exposures from the 25th to the 50th percentile was below zero; however, there was a steady increase from the 55th percentile to the 75th percentile for triglyceride, total cholesterol, LDL cholesterol, non-HDL cholesterol, and diastolic blood pressure. For HDL cholesterol and systolic blood pressure, there was a steady increase from the 65th to 75th percentile. This trend underscores the importance of monitoring and managing combined exposures to toxic metals and essential elements to mitigate the risk of CVD, particularly as exposure levels increase beyond certain thresholds.

### 3.4. Quantile G-Computation

**Figure 7:**
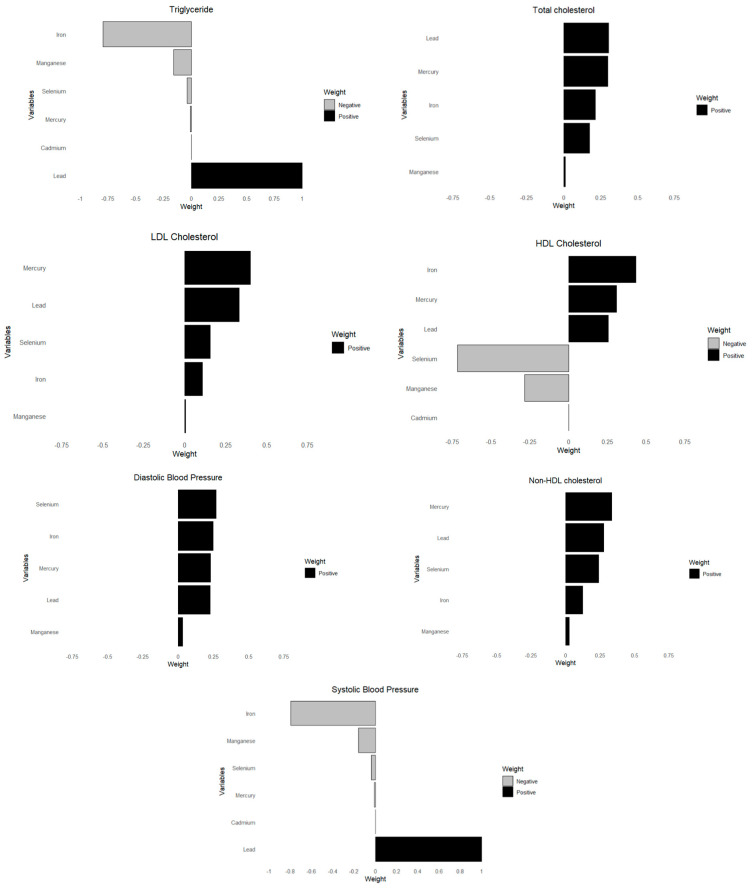
Weights of each toxic metal and essential element on CVD outcome using quantile G-computation. Adjusted for age, sex, race/ethnicity, BMI, income, smoking status, and alcohol use.

This analysis investigated the associations between toxic metals and essential minerals on cardiovascular risk markers, including triglycerides, total cholesterol, LDL cholesterol, HDL cholesterol, non-HDL cholesterol, diastolic blood pressure, and systolic blood pressure. Using quantile g-computation (Figure 7), we derived weights that reflect both the direction and relative strength of each exposure’s impact on these cardiovascular outcomes.

### 3.5. Weighted Quantile Sum Regression (WQSR)

**Figure 8:**
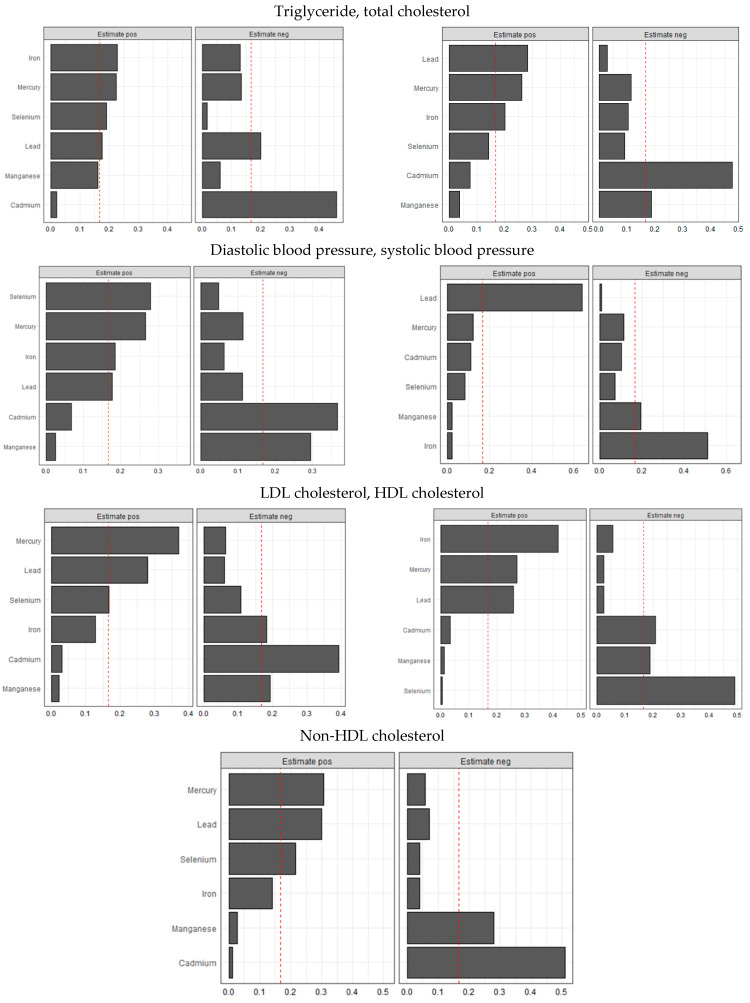
Weighted quantile sum regression analysis of toxic metals and essential elements on CVD. Adjusted for age, sex, race/ethnicity, BMI, income, smoking status, and alcohol use.

Figure 8 below shows WQSR analyses, examining the influences of toxic metals and essential elements on different CVD biomarkers. These biomarkers include triglycerides, total cholesterol, LDL cholesterol, HDL cholesterol, non-HDL cholesterol, diastolic blood pressure, and systolic blood pressure.

## 4. Discussion

### 4.1. Correlation Analysis

The Spearman correlation analysis revealed strong correlations among toxic metals, whereas essential elements exhibited weaker intercorrelations. This suggests that toxic metals often co-occur in environmental exposures [35], potentially compounding their effects on cardiovascular disease (CVD) risk factors.

### 4.2. Bayesian Kernel Machine Regression (BKMR) Analysis

The BKMR analysis identified lead, mercury, iron, and selenium as consistently demonstrating high posterior inclusion probability (PIP) values across nearly all CVD risk factors. Cadmium was strongly associated with HDL cholesterol, diastolic blood pressure, and systolic blood pressure, while manganese showed significant associations with LDL and HDL cholesterol. Furthermore, conditional probability analysis highlighted lead and selenium as the most influential elements within their respective groups, exhibiting strong associations with nearly all CVD markers. These findings underscore the critical impact of both toxic metals and essential elements on CVD risk. This finding reinforces previous research indicating that lead exposure can impair lipid metabolism, while selenium deficiency may exacerbate dyslipidemia [36,37,38,39,40]. Notably, while essential elements have protective effects, their interactions with toxic metals may modify their influence.

### 4.3. Univariate and Bivariate Analysis

The univariate analysis revealed that lead exhibited strong and complex associations with triglycerides, non-HDL-C, SBP, and DBP levels, while selenium (LDL cholesterol) and iron (HDL cholesterol, non-HDL-C, LDL-C) were more closely linked to cholesterol-related outcomes. The bivariate approach further captured these interactions, particularly those involving lead, and their associations across multiple CVD outcomes.

These findings confirm that toxic metal exposure is significantly associated with adverse cardiovascular outcomes, and essential elements may mitigate some of these risks. Lead demonstrated complex relationships with total cholesterol, LDL cholesterol, non-HDL cholesterol, and both diastolic and systolic blood pressure, reaffirming the need for advanced statistical techniques to fully capture these intricate relationships. Many of these results align with the existing literature, where lead exposure has been linked to an increased risk of hypertension and atherosclerosis through oxidative stress and endothelial dysfunction [41]. Similarly, mercury exhibited strong associations with triglycerides, supporting previous studies that connect mercury exposure to disrupted lipid metabolism and cardiovascular dysfunction [42].

### 4.4. Single-Variable Effects at Increasing Quantiles

The single-variable effects of toxic metals and essential elements at increasing quantiles of all other exposures provided further insight into these complex relationships.

For triglycerides, shifting iron from the 0.75 quantile to the 0.25 quantile while moving all other exposures from 0.25 to 0.50 to 0.75 indicated notable interactions. Additionally, mercury, cadmium, selenium, and manganese exhibited significant interactions for triglyceride levels.

For total cholesterol, iron demonstrated significant interaction under this scenario, as did manganese, mercury, and lead. In contrast, very little interaction was observed for LDL cholesterol.

For HDL cholesterol, manganese exhibited the most significant interaction when compared to all other exposures. Cadmium and lead, and to a lesser extent, selenium, mercury, and iron also showed interactions.

For non-HDL cholesterol, mercury, selenium, and lead displayed significant interactions, with cadmium and iron also showing some degree of interaction.

For diastolic blood pressure, selenium exhibited significant interaction, as did lead, with iron and manganese showing some but less extensive interaction.

For systolic blood pressure, manganese showed interaction, along with selenium, mercury, and cadmium, although their interactions were minimal.

Collectively, these results indicate that both toxic metals and essential elements interact in complex ways to influence lipid metabolism and blood pressure regulation. The observed interactions, particularly for iron, lead, mercury, cadmium, selenium, and manganese, suggest that metal exposures may modulate cardiovascular risk differently across lipid fractions and blood pressure parameters. Prior studies have demonstrated that heavy metals are linked to altered cholesterol profiles [43], increased triglycerides [44], and hypertension [45], with some essential elements playing protective or modifying roles [46,47].

These findings highlight the critical importance of evaluating interactions rather than considering pollutants in isolation. Given the co-exposure nature of environmental contaminants, single-metal approaches may overlook key synergistic or antagonistic effects that shape disease risk. Interactions between toxic and essential elements can amplify or mitigate adverse health effects, influencing lipid regulation and blood pressure through shared biological pathways. Understanding these complex relationships is essential for refining risk assessments, developing targeted public health interventions, and improving regulatory guidelines to account for the multifaceted impact of environmental exposures.

### 4.5. Quantile g-Computation and Weighted Quantile Sum Regression (WQSR) Analysis

Quantile g-computation highlighted the relative weights of each toxic metal and essential element, with lead consistently demonstrating a strong contribution to the mixture across almost all cardiovascular-related outcomes. These findings underscore lead’s pervasive impact on lipid metabolism and blood pressure regulation, aligning with prior studies that have linked chronic lead exposure to hypertension, atherosclerosis, and dyslipidemia through oxidative stress and endothelial dysfunction. The associations of other metals and essential elements varied depending on the specific outcomes, indicating complex interactive effects within the mixture. For instance, selenium exhibited a protective role in HDL cholesterol regulation, while manganese showed both protective and detrimental associations depending on the exposure quantile and outcome measured. Cadmium and mercury, known for their toxic cardiovascular effects, contributed significantly to triglyceride and blood pressure outcomes, reinforcing the existing literature that implicates these metals in metabolic and vascular dysregulation. The patterns observed in quantile g-computation were similarly reflected in the weighted quantile sum regression (WQSR) analysis, further validating the importance of mixture modeling in capturing these nuanced exposure–response relationships.

### 4.6. Public Health Implications

The results from the BKMR, Qgcomp, and WQSR provide robust insights into the intricate interactions between environmental exposures and CVD health outcomes. Lead’s consistent and strong association with most CVD biomarkers suggests that it is a key factor in CVD risk, a finding supported by previous research [32]. Additionally, our study highlights the distinct effects of toxic metals and essential elements on lipid profiles and blood pressure, reinforcing the role of oxidative stress, inflammation, disrupted lipid metabolism, as well as potential altered cholesterol transport in CVD development.

These findings underscore the need for public health interventions aimed at reducing toxic metal exposure, particularly among vulnerable populations. Environmental exposure to lead, cadmium, and mercury disproportionately affects low-income and minority communities, necessitating targeted policies to mitigate these risks. Strategies such as stricter environmental regulations on industrial emissions, improved water filtration systems, and nutritional interventions to enhance essential element intake could help offset the cardiovascular risks posed by toxic metals.

Furthermore, the protective roles of selenium, manganese, and iron suggest that dietary interventions may serve as a mitigating factor against heavy metal toxicity. Nutritional supplementation programs, particularly in populations with high metal burdens, could help reduce the adverse cardiovascular effects of toxic metal exposure. These findings emphasize that while essential elements provide health benefits, their interactions with toxic metals may modulate their effectiveness, reinforcing the need for exposure reduction strategies that consider both individual toxicants and broader environmental and dietary influences on cardiovascular health.

### 4.7. Strengths and Limitations

A key strength of this study is the application of advanced mixture modeling techniques, which provide a more comprehensive understanding of the relationships between multiple exposures and cardiovascular outcomes. By utilizing BKMR, WQSR, and quantile g-computation, this study assessed the independent, combined, and interactive effects of toxic metals and essential elements on CVD risk markers.

However, some limitations should be acknowledged. The cross-sectional nature of the NHANES dataset precludes causal inference, necessitating longitudinal studies to confirm causal relationships. Additionally, while major confounders such as age, sex, race/ethnicity, BMI, and socioeconomic factors were adjusted for, residual confounding due to unmeasured variables cannot be ruled out. Finally, while NHANES provides a representative sample of the U.S. population, findings may not be generalizable to regions with different environmental and dietary exposures.

### 4.8. Future Directions

Future research should explore longitudinal associations between metal exposure and CVD progression. Mechanistic studies investigating the interactions of toxic metals and essential elements at the molecular level could further elucidate their roles in CVD pathogenesis. Additionally, intervention studies examining dietary or pharmacological strategies to mitigate metal-related cardiovascular risks would be highly valuable.

## 5. Conclusions

This study enhances our understanding of the complex interactions and mechanisms through which environmental metals may impact the risk of CVD. It highlights the value of using BKMR, quantile g-computation, and WQSR in environmental health research and emphasizes the importance of investigating combined and individual exposures to toxic metals and essential elements in relation to critical health outcomes. Additionally, it addresses gaps in the literature on the health effects of multiple environmental pollutants, providing critical results that can eventually lead to a comprehensive approach to exposure assessment and risk management of exposures to safeguard public health.

## Figures and Tables

**Table 1:** Descriptive statistics for blood concentrations of toxic metals, essential elements, and cardiovascular biomarkers.

(**a**)									
**Variable**		**Mean (** **SD)**		**Minimum**				**Maximum**	
**Toxic metals**									
Lead		1.06 (1.35)		0.00				42.00	
Cadmium		0.25 (0.59)		0.00				13.00	
Mercury		1.07 (2.33)		0.00				64.00	
**Essential elements**									
Selenium		186.30 (26.34)		85.00				454.00	
Manganese		10.32 (3.77)		2.00				52.00	
Iron		87.28 (36.62)		10.00				476.00	
**CVD Biomarkers**									
Triglyceride		107.34 (98.26)		10.00				2684.00	
Total Cholesterol		179.89 (40.60)		76.00				446.00	
LDL Cholesterol		108.50 (36.33)		20.00				359.00	
HDL Cholesterol		53.39 (14.75)		10.00				189.00	
Non-HDL Cholesterol		126.50 (40.57)		22.00				408	
Diastolic Blood Pressure		67.79 (15.55)		0.00				135.00	
Systolic Blood Pressure		120.83 (19.55)		73.00				225.00	
(**b**) Demographic characteristics, body mass index (BMI), and alcohol use in the study population									
**Variables**	**Description**		**Frequency**		**Mean**	**Percentage**	**95% Confidence Interval**		
**Gender**			9254						
	Male		4557		37.43	49.24	36.38		38.48
	Female		4697		39.38	50.76	38.12		40.64
**Ethnicity**			9254						
	Mexican American		1367			14.77	27.50		31.13
	Other Hispanic		820			8.86	31.77,		35.60
	Non-Hispanic White		3150			34.04	40.00,		43.34
	Non-Hispanic Black		2115			22.85	34.68		36.98
	Non-Hispanic Asian		1168			12.62	35.93		39.93
	Other Races—Including Multi-Racial		634			6.85	29.59		37.36
**BMI**	Male		8878		27.45		26.91		28.00
	Female		8931		27.45		27.21		28.55
**Alcohol Use**			5130						
	Yes		4545			88.60			
	No		585			11.49			

**Table 2:** Posterior inclusion probabilities for the influence of toxic metals and essential elements on CVD.

	Triglyceride	Total Cholesterol	LDL Cholesterol	HDL Cholesterol	Non-HDL Cholesterol	Diastolic Blood Pressure	Systolic Blood Pressure
Lead	0.3022	1.0000	0.9564	1.0000	1.0000	1.0000	1.0000
Cadmium	0.3106	0.0000	0.5020	1.0000	0.2528	0.9612	0.9496
Mercury	0.9782	1.0000	0.9296	0.9892	1.0000	0.4828	0.3608
Selenium	0.9838	1.0000	1.0000	0.9992	1.0000	1.0000	0.2688
Manganese	0.3314	0.2524	0.6556	1.0000	0.2912	0.2192	0.3692
Iron	0.9636	1.0000	0.8028	1.0000	1.0000	0.9900	0.0148

**Table 3:** Hierarchical BKMR results for triglyceride.

	Group	Group PIP	Cond PIP
Lead	1	0.8900	0.0418
Cadmium	1	0.8900	0.0674
Mercury	1	0.8900	0.8908
Selenium	2	0.9852	0.7353
Manganese	2	0.9852	0.0057
Iron	2	0.9852	0.2590

**Table 4:** Hierarchical BKMR results for total cholesterol.

	Group	Group PIP	Cond PIP
Lead	1	1.0000	1.0000
Cadmium	1	1.0000	0.0000
Mercury	1	1.0000	0.0000
Selenium	2	1.0000	1.0000
Manganese	2	1.0000	0.0000
Iron	2	1.0000	0.0000

**Table 5:** Hierarchical BKMR results for LDL cholesterol.

	Group	Group PIP	Cond PIP
Lead	1	1.0000	0.9556
Cadmium	1	1.0000	0.0000
Mercury	1	1.0000	0.0444
Selenium	2	1.0000	0.8156
Manganese	2	1.0000	0.0000
Iron	2	1.0000	0.1844

**Table 6:** Hierarchical BKMR results for HDL cholesterol.

	Group	Group PIP	Cond PIP
Lead	1	1.0000	1.0000
Cadmium	1	1.0000	0.0000
Mercury	1	1.0000	0.0000
Selenium	2	1.0000	0.0000
Manganese	2	1.0000	0.0000
Iron	2	1.0000	1.0000

**Table 7:** Hierarchical BKMR results for non-HDL cholesterol.

	Group	Group PIP	Cond PIP
Lead	1	1.0000	1.0000
Cadmium	1	1.0000	0.0000
Mercury	1	1.0000	0.0000
Selenium	2	1.0000	1.0000
Manganese	2	1.0000	0.0000
Iron	2	1.0000	0.0000

**Table 8:** Hierarchical BKMR results for diastolic blood pressure.

	Group	Group PIP	Cond PIP
Lead	1	1.0000	1.0000
Cadmium	1	1.0000	0.0000
Mercury	1	1.0000	0.0000
Selenium	2	1.0000	1.0000
Manganese	2	1.0000	0.0000
Iron	2	1.0000	0.0000

**Table 9:** Hierarchical BKMR results for systolic blood pressure.

	Group	Group PIP	Cond PIP
Lead	1	1.0000	1.0000
Cadmium	1	1.0000	0.0000
Mercury	1	1.0000	0.0000
Selenium	2	1.0000	0.3208
Manganese	2	1.0000	0.4025
Iron	2	1.0000	0.2767

## Data Availability

The NHANES dataset is publicly available online, accessible at https://wwwn.cdc.gov/nchs/nhanes/continuousnhanes/overview.aspx?BeginYear=2017 (accessed on 1 February 2025).
